# The Value of ADAMTS13 in Predicting Clinical Outcomes in Patients With Acute Ischemic Stroke Receiving Thrombolysis

**DOI:** 10.3389/fneur.2020.00799

**Published:** 2020-07-31

**Authors:** Ya Su, Xin Chen, Xiaofei Ye, Haiyan Sun, Fei Wu, Qiang Dong, Xin Cheng, Danhong Wu

**Affiliations:** ^1^State Key Laboratory of Medical Neurobiology, Department of Neurology, National Clinical Research Centre for Aging and Medicine, Huashan Hospital, Fudan University, Shanghai, China; ^2^Department of Neurology, Shanghai Fifth People's Hospital, Fudan University, Shanghai, China; ^3^Department of Health Statistics, Second Military Medical University, Shanghai, China; ^4^Institute of Hematology & Blood Diseases Hospital, Chinese Academy of Medical Sciences, Tianjin, China

**Keywords:** ADAMTS13, ischemic stroke, thrombolysis, clinical outcome, hemorrhagic transformation, infarct core, collateral flow

## Abstract

**Objective:** To determine the association between baseline ADAMTS13 (a disintegrin and metalloproteinase with a thrombospondin type 1 motif, member 13) antigen level and 90-days clinical outcome in patients with acute ischemic stroke (AIS) receiving recombinant tissue plasminogen activator (rt-PA) thrombolysis.

**Methods:** AIS patients receiving rt-PA thrombolytic therapy from Huashan Hospital and Fifth People's Hospital of Shanghai, China in 2014–2017 were consecutively enrolled. Blood samples for ADAMTS13 tests were drawn before intravenous rt-PA administration. The primary outcome was defined as the poor functional outcome of modified Rankin Scale (mRS) >2 at 90-days follow-up. Secondary outcome was hemorrhagic transformation after rt-PA therapy. Moreover, for AIS patients with large vessel occlusion from Huashan Hospital, the association between baseline ADAMTS13 level and cerebral collateral flow was also assessed.

**Results:** A total of 163 AIS patients (median age 66.2 years, 63.8% male) were included. Baseline ADAMTS13 level was marginally decreased in patients with 90-days mRS >2 than in those with mRS ≤ 2 (mean ± SD, 1458.4 ± 323.3 vs. 1578.3 ± 395.4 ng/mL, *p* = 0.046). However, no difference of ADAMTS13 level was found after adjusting for age, history of atrial fibrillation, glycemia, baseline NIHSS score and TOAST classification (*p* = 0.43). We found no difference in ADAMTS13 level between patients with parenchymal hemorrhage after rt-PA therapy and those without (*p* = 0.44). Among 66 patients with large vessel occlusion, there was also no association between ADAMTS13 level and cerebral collateral flow in multivariable analyses.

**Conclusion:** In our cohort, blood ADAMTS13 antigen level before rt-PA therapy could not be used as an independent biomarker in predicting clinical outcomes of AIS patients at 90 days.

## Introduction

Intravenous thrombolysis with recombinant tissue plasminogen activator (rt-PA) within 4.5 h after the onset of stroke has been the major treatment for acute ischemic stroke (AIS) (1). It is strongly associated with an increased probability of survival without handicap and dependency at 3 months after ischemic stroke (2, 3). However, rt-PA treatment may lead to distinct clinical outcomes and also increase the risk of symptomatic intracerebral hemorrhage (sICH) (4, 5). Therefore, discovery of a blood biomarker for predicting prognosis and sICH is of great interest and help.

Previous studies have shown that von Willebrand factor (VWF) plays a vital role in thrombus formation at sites of vascular damage (6, 7). VWF activities are associated with its multimer size, with ultra-large multimers spontaneously binding to platelets (8). Interestingly, ADAMTS13 (a disintegrin and metalloproteinase with a thrombospondin type 1 motif, member 13) is a protease that can cleave ultra-large VWF into smaller, less reactive multimers, and the deficiency of ADAMTS13 can cause thrombotic occlusion of micro-vessels from multiple organs, including the brain (9). The VWF-ADAMTS13 axis has been proven to play a significant role in the pathophysiological microcirculatory disturbance of ischemic stroke (10). In animal studies, absence of ADAMTS13 exacerbates outcomes of ischemia or reperfusion injury (11, 12), while injecting recombinant ADAMTS13 reduces rt-PA-associated hemorrhage (13). Plasma VWF levels are elevated in diseases associated with the blood-brain barrier disruption (14), while whether low ADAMTS13 level is associated with more rt-PA-induced hemorrhage is unknown. In AIS patients treated with rt-PA, ADAMTS13 can predict recanalization, with no effect on functional outcome 3 months after stroke (15, 16). Its relationship with thrombus extension promotion might also play a role in the collateral flow after AIS.

In this study, we aimed to examine whether low ADAMTS13 antigen level before rt-PA treatment (1) could predict functional outcomes 90 days after stroke; (2) could predict hemorrhagic transformation; (3) was associated with poor collateral flow in a subset of AIS patients with large vessel occlusion.

## Materials and Methods

### Study Population and Imaging Processing

We prospectively recruited consecutive AIS patients receiving rt-PA thrombolytic therapy from Huashan Hospital and Fifth People's Hospital of Shanghai, China from November 2014 to November 2017. The inclusion criteria included: (1) available blood samples after stroke onset and before rt-PA therapy; (2) underwent computed tomography (CT) scan at 24 h and magnetic resonance imaging (MRI) within 7 days; (3) complete baseline clinical evaluation including National Institutes of Health Stroke Scale (NIHSS) and follow-up data; (4) patients with mRS ≤ 2 prior to stroke. All patients were treated with intravenous rt-PA according to guidelines and clinical judgment of acute stroke teams. The primary outcome was defined as the poor functional outcome at 90-days follow-up (modified Rankin Scale [mRS] > 2), which was recorded by a trained neurologist blinded to patient baseline information using a validated telephone script. Secondary outcomes included parenchymal hemorrhage (PH), especially PH Type 2 (PH2), on CT scan after 24 h and at day 7 after rt-PA therapy as defined in the European Cooperative Acute Stroke Study II (4). The study was approved by local ethics committees and the written informed consent was provided by the patient or authorized family members.

For AIS patients with large vessel occlusion from Huashan Hospital, we further assessed the collateral flow in the acute phase as previously reported (17). Briefly, the patients underwent complete baseline multimodal CT imaging including CT Angiography (CTA) and CT perfusion (CTP). The volumes of acute hypoperfused lesion (delay time [DT] > 3 s), severely hypoperfused lesion (DT > 6 s) and infarct core (relative cerebral blood flow [rCBF] <30%) were calculated by validated thresholds.

### Blood Samples and ADAMTS13 Measurements

Experimenters were all blinded to the clinical and imaging data. Peripheral blood (2 ml) was drawn with promoting coagulating tubes in the emergency room before rt-PA treatment and were centrifuged at 3,000 rpm for 15 min within 4 h. Then the serums were stored at −80°C until analysis. We measured the ADAMTS13 antigen level by ELISA using commercially available kits (LXSAHM, Magnetic Luminex Screening Assay, Human Premixed Multi-Analyte Kit; R&D Systems, MN) per manufacturer instructions. Intra-assay variabilities were <15%.

### Statistical Analysis

Statistical analyses were performed using SPSS version 20.0 (SPSS Inc., Chicago, IL). A two-tailed *P* < 0.05 was considered as significant. Continuous variables were described as mean ± standard deviation (SD) (for normal distributions) or median with interquartile range (IQR) (for non-normal distributions) and categorical variables were expressed as counts with percentages (*n* [%]). We compared clinical characteristics and ADAMTS13 level between groups with mRS ≤ 2 vs. mRS >2, and groups with vs. without PH, using the Student *t*-test or Mann-Whitney test for continuous variables and the χ^2^ or Fisher exact test for categorical variables. In further multivariable analyses, sex, age and all covariables associated with *p* < 0.05 in the univariable analysis were included. Univariable and multivariable logistic regression models were used to estimate the unadjusted or adjusted odds ratio (OR) with 95% confidence interval (CI) on the poor outcome at 3 months and PH per ADAMTS13 level. For subgroup analyses of patients with large vessel occlusion and CTP examination, the association between ADAMTS13 level and collateral flow was evaluated using univariable and multivariable linear models (coefficient [95% CI]).

## Results

A total of 163 patients (mean age 66.2 ± 12.7 years, 63.8% men) were recruited in our study, 102 patients from Huashan Hospital and 61 from Fifth People's Hospital of Shanghai.

### ADAMTS13 and Functional Outcome

In the whole cohort, baseline ADAMTS13 level was marginally decreased in patients with mRS >2 at 90 days than in those with mRS ≤2 (1458.4 ± 323.3 vs. 1578.3 ± 395.4 ng/mL, *p* = 0.046). Meanwhile, patients with poor outcomes were older (*p* = 0.001), with more history of atrial fibrillation (*p* < 0.001), higher level of blood glycemia (*p* = 0.002) and baseline NIHSS score (*p* < 0.001), and showed a smaller percentage of small artery occlusion but more cardio-embolism (*p* < 0.001) (Table 1). In further multivariable logistic regression analysis, no difference of ADAMTS13 level was found after adjusting for sex and age (OR: 0.88 [0.75–1.03], *p* = 0.12), and all the confounding factors (OR: 0.92 [0.75–1.13], *p* = 0.43) (Table 2).

**Table 1:** Baseline characteristics of the study patients with acute ischemic stroke receiving rt-PA thrombolytic therapy, according to mRS status.

**Parameter**	**Total (*n* = 163)**	**mRS 0–2 (*n* = 101)**	**mRS >2 (*n* = 62)**	***p*-value**
Age, mean ± SD, years	66.2 ± 12.7	63.7 ± 10.9	70.3 ± 14.3	0.001
Sex, male, *n* (%)	104 (63.8)	69 (68.3)	35 (56.5)	0.13
Hypertension, *n* (%)	101 (62.0)	59 (58.4)	42 (67.8)	0.23
Diabetes mellitus, *n* (%)	45 (27.6)	25 (24.8)	20 (32.3)	0.30
Hyperlipidemia, *n* (%)	36 (22.1)	27 (26.7)	9 (14.5)	0.07
Smoking, *n* (%)	63 (38.7)	38 (37.6)	25 (40.3)	0.73
Atrial fibrillation, *n* (%)	36 (22.1)	13 (12.9)	23 (37.1)	<0.001
Previous stroke, *n* (%)	21 (12.9)	13 (12.9)	8 (12.9)	1.00
Antiplatelets, *n* (%)	29 (17.8)	14 (13.9)	15 (24.2)	0.09
Anticoagulation, *n* (%)	11 (6.7)	5 (5.0)	6 (9.7)	0.34
SBP, median (IQR), mmHg	150 (135, 164)	150 (139, 163)	142 (134, 165)	0.29
DBP, median (IQR), mmHg	85 (77, 95)	84 (77, 95)	89 (76, 98)	0.50
Glycemia, median (IQR), mg/dL	122 (103, 155)	115 (98, 140)	136 (111, 180)	0.002
Baseline NIHSS score, median (IQR)	6 (3–13)	4 (2–7)	13 (8–18.3)	<0.001
Onset-to-tPA, median (IQR), min	175 (125–220)	180 (135–222)	163 (120–195)	0.06
**TOAST classification**, ***n*** **(%)**				
LAA	72 (44.2)	44 (43.6)	28 (45.2)	<0.001
SAO	26 (16.0)	24 (23.8)	2 (3.2)	
CE	46 (28.2)	19 (18.8)	27 (43.5)	
SOD and SUD	19 (11.7)	14 (13.9)	5 (8.1)	
Abnormal C-reactive protein, *n* (%)	12 (7.4)	5 (5.0)	7 (11.3)	0.22
ADAMTS13, mean ± SD, ng/mL	1532.6 ± 373.2	1578.3 ± 395.4	1458.4 ± 323.3	0.046

*mRS, modified Rankin Scale; SBP, systolic blood pressure; DBP, diastolic blood pressure; rt-PA, recombinant tissue plasminogen activator; IQR, interquartile range; TOAST, Trial of org 10,172 in Acute Stroke Treatment; LAA, Large-artery atherosclerosis; SAO, Small-artery occlusion; CE, Cardio-embolism; SOD, Stroke of other determined cause; SUD, Stroke of undetermined cause; ADAMTS13, a disintegrin and metalloproteinase with a thrombospondin type 1 motif, member 13*.

**Table 2:** The association between ADAMTS13 level and clinical outcomes and parenchymal hemorrhage.

**ADAMTS13 (per 100 ng/mL)**	**Unadjusted**		**Sex- and age-adjusted**		**Multivariable adjusted**^****a****^	
	**OR (95% CI)**	***p*-value**	**OR (95% CI)**	***p*-value**	**OR (95% CI)**	***p*-value**
mRS 0–2	0.86 (0.74–0.999)	0.049	0.88 (0.75–1.03)	0.12	0.92 (0.75–1.13)	0.43
PH	1.09 (0.88–1.34)	0.44	1.08 (0.87–1.35)	0.48	1.17 (0.92–1.50)	0.20
PH 2	0.94 (0.70–1.27)	0.69	0.93 (0.68–1.28)	0.66	1.02 (0.74–1.40)	0.92

*ADAMTS13, a disintegrin and metalloproteinase with a thrombospondin type 1 motif, member 13; OR, odds ratio; CI, Confidence interval; PH, parenchymal hemorrhage*.

a *Adjusted for age, history of atrial fibrillation, glycemia, baseline NIHSS score and TOAST classification*.

### ADAMTS13 and PH After rt-PA

**Figure 1:**
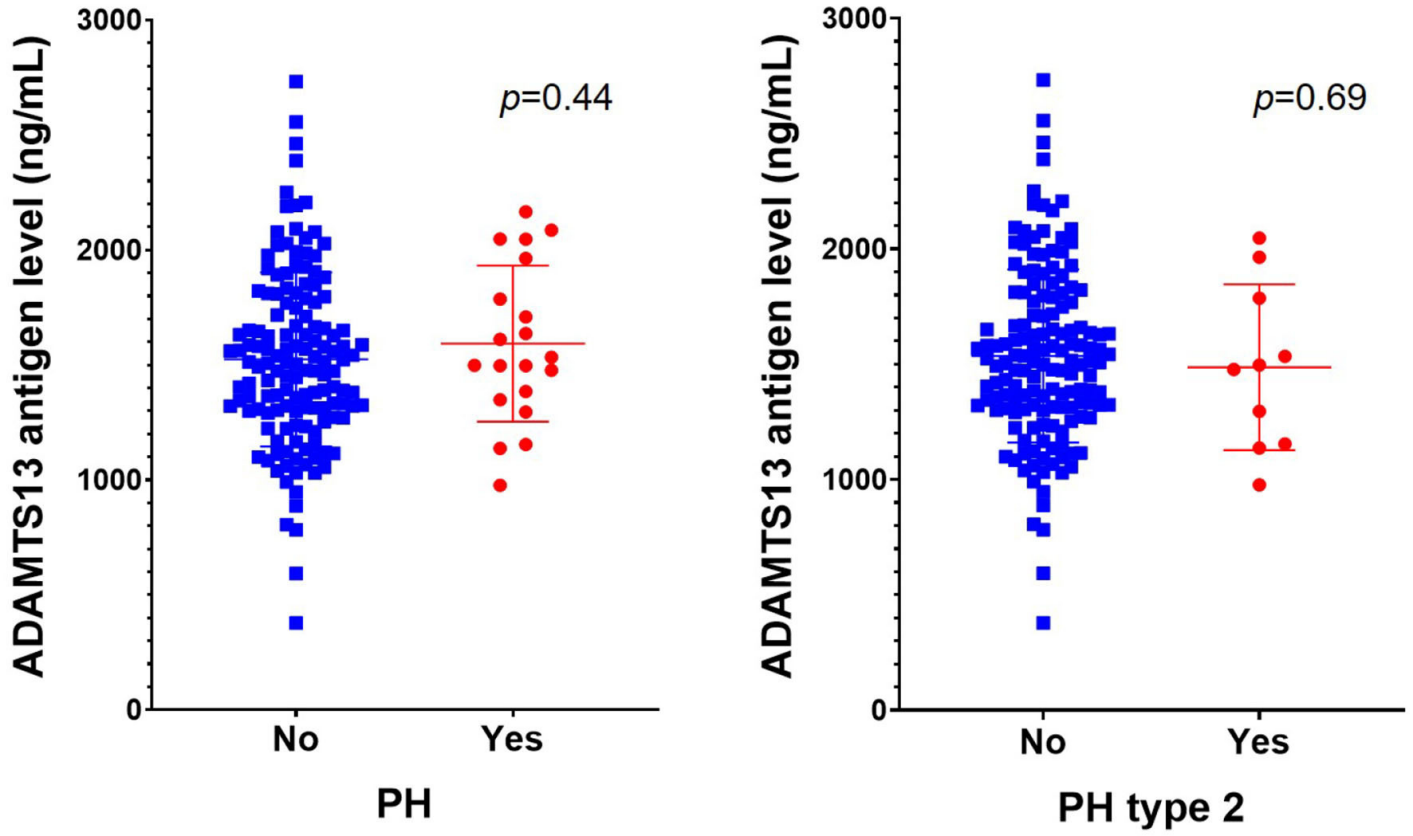
Comparison of ADAMTS13 level between AIS patients with and without intracranial PH after rt-PA therapy. Data are expressed as mean ± SD. The ADAMTS13 levels were 1524.2 ± 378.1 and 1592.6 ± 339.5 ng/mL in patients without and with PH, and 1535.6 ± 375.1 and 1486.9 ± 359.3 ng/mL in patients without and with PH2 after rt-PA, respectively. ADAMTS13, a disintegrin and metalloproteinase with a thrombospondin type 1 motif, member 13; AIS, acute ischemic stroke; PH, parenchymal hemorrhage; rt-PA, recombinant tissue plasminogen activator.

There were 27 patients with hemorrhagic transformation after rt-PA therapy, 20 of them with PH type (10 with PH2 type). There were no differences of ADAMTS13 level between patients with PH after rt-PA therapy and those without (1592.6 ± 339.5 vs. 1524.2 ± 378.1 ng/mL, *p* = 0.44), or patients with PH2 and those without (1486.9 ± 359.3 vs. 1535.6 ± 375.1 ng/mL, *p* = 0.69) (Figure 1). Similar results were obtained after adjusting for sex and age (for PH: OR: 1.08 [0.87–1.35], *p* = 0.48; for PH2: (OR: 0.93 [0.68–1.28], *p* = 0.66), or adjusting age, history of atrial fibrillation, glycemia, baseline NIHSS score and TOAST classification (for PH: OR: 1.17 [0.92–1.50], *p* = 0.20; for PH2: (OR: 1.02 [0.74–1.40], *p* = 0.92) (Table 2).

### ADAMTS13 and Collateral Flow

In 66 patients with large vessel occlusion, there was no association of ADAMTS13 level and collateral flow (for core volume: coefficient: 0.25 [−3.86–4.37], *p* = 0.90), for DT > 3 s: coefficient: −1.58 [−9.00–5.84], *p* = 0.67; for DT > 3 s/DT > 6 s: coefficient: −0.47 [−1.89–0.96], *p* = 0.51) in multivariable analyses (Table 3).

**Table 3:** The association between ADAMTS13 level and collateral flow parameters in patients with large vessel occlusion and baseline multimodal computed tomography.

**ADAMTS13 (per 100 ng/mL)**	**Unadjusted**		**Sex- and age-adjusted**		**Multivariable adjusted**^****a****^	
	**Coefficient (95% CI)**	***p*-value**	**Coefficient (95% CI)**	***p*-value**	**Coefficient (95% CI)**	***p*-value**
Core volume	−2.78 (−6.87 to 1.31)	0.18	−1.87 (−6.08 to 2.33)	0.38	0.25 (−3.86 to 4.37)	0.90
DT > 3 s	−9.68 (−17.9 to −1.44)	0.022	−6.85 (−15.0 to 1.29)	0.10	−1.58 (−9.00 to 5.84)	0.67
DT > 3 s/DT > 6 s	−0.01 (−1.32 to 1.30)	0.99	−0.18 (−1.51 to 1.16)	0.80	−0.47 (−1.89 to 0.96)	0.51

*ADAMTS13, a disintegrin and metalloproteinase with a thrombospondin type 1 motif, member 13; CI, Confidence interval; DT, delay time*.

a *Adjusted for age, history of atrial fibrillation and baseline NIHSS score*.

## Discussion

In the present study, we examined the antigen level of ADAMTS13 before thrombolysis in 163 AIS patients and found the ADAMTS13 level was lower in patients with mRS >2 at 90 days, but it was not independently associated with the outcome after adjusting for all confounding factors. Meanwhile, there was no significant difference between patients with and without PH after rt-PA therapy. In the subgroup analysis (among 66 patients with large vessel occlusion and baseline CTP evaluation), we also found no independent associations between ADAMTS13 level and cerebral collateral flow parameters.

The dysregulated VWF-ADAMTS13 axis has great effects on ischemic stroke (10). Bustamante et al. (15) revealed that reduced ADAMTS13 activity level was related to poor response to recanalization therapies (both in patients treated with rt-PA and mechanical thrombectomy) and Putzer et al. (16) found that the lowest quartile of ADAMTS13 activity was independently associated with less early improvement in NIHSS score (OR 1.298, *p* = 0.050). There was no association of ADAMTS13 level and 90-days functional outcomes in both studies, which was consistent with our finding. However, another study (18) of AIS patients treated with endovascular treatment found that low ADAMTS13 activity level was independently associated with unfavorable outcomes (mRS > 4). The different results might be caused by several reasons. First, interventional therapy could improve the outcome by providing a higher likelihood of achieving successful reperfusion and a lower chance of complications (19, 20). Second, researchers in this study considered mRS > 4 as the unfavorable outcome at 90 days which was different from our study (mRS > 2). In addition, we measured the level of ADAMTS13 antigen, which was different from the above studies. These reasons might affect the grouping that was eventually included in the analysis.

Animal studies showed that the recovery of brain tissue in ADAMTS13^−/−^ mice was significantly lower than that in wild-type mice at 14 days after ischemic stroke (21) and intraventricular injection of recombinant ADAMTS13 3 h after middle cerebral artery occlusion could reduce tPA-induced hemorrhage by regulating blood brain barrier integrity (13). However, in our AIS patients, the ADAMTS13 antigen level was not independently associated with PH after rt-PA therapy. It has been reported that ADAMTS13 activity could return to normal level in the late phase (≥3 months) of ischemic stroke (22). There was a dynamic change of ADAMTS13 activity level in human during the process of AIS. The level of ADAMTS13 antigen might have similar changes, but blood samples in our study were collected just before rt-PA thrombolysis. This was different when compared with ADAMTS13^−/−^ mice. Moreover, ADAMTS13 level of the blood might not vary synchronously with that of the infarct or ischemic area in the brain. Whether ADAMTS13 treatment could be expected as a new therapeutic target still need further clinical studies.

Also from animal studies, the cerebral infarct volume was significantly increased with lower ADAMTS13 level after ischemic stroke (23) and regional blood flow of the ischemic cortex after reperfusion was significantly decreased in ADAMTS13^−/−^ mice than that in wild-type mice (24). Therefore, we hypothesized that decreased ADAMTS13 might be associated with poor collateral flow in AIS patients with large vessel occlusion. Our previous study demonstrated that core volume, DT > 3 s and DT > 3 s/DT > 6 s could be used as parameters for collateral flow in AIS patients (17). However, we didn't find the independent association between ADAMTS13 level and collateral flow in our patients. Thus, ADAMTS13 might not influence recanalization or clinical outcomes through altering collateral flow.

This study had some limitations. First, although this was a double-center study, the sample size was relatively small especially the size of patients who received perfusion examination in the acute phase, which might cause higher selection biases. Meanwhile, the number of patients with hemorrhagic transformation might also be relatively small. Large pre-specified patient cohort will be needed to further confirm the conclusion. Second, we only collected blood samples before rt-PA thrombolysis. Since ADAMTS13 level varied over time after stroke process, serial blood samples will be needed to determine which timepoint was the best for predicting long-term clinical outcomes and hemorrhagic transformation. Third, the ADAMTS13 activity can only be measured in citrated plasma and such samples were not routinely collected for AIS patients. Since the activity assay had intrinsic limitations that it was performed in static conditions but had higher variabilities *in vitro* and in different pathologic conditions (15, 25), we chose the ADAMTS13 antigen level for more reliable and reproducible results. However, the utility of ADAMTS13 antigen was also controversial. In future studies, the ADAMTS13 antigen and activity, the VWF antigen and multimer size, and other molecules involved in the ADAMTS13/VWF axis should be simultaneously compared to confirm the utility of ADAMTS13 in AIS.

In conclusion, our study showed that ADAMTS13 level before rt-PA thrombolysis was decreased in AIS patients with 90-days mRS >2, but was not an independent predictor for clinical outcomes. There was also no independent association between ADAMTS13 level and hemorrhagic transformation or collateral flow. Therefore, blood ADAMTS13 level could not be used as an independent biomarker in predicting clinical outcomes of AIS patients at 90 days.

## Data Availability Statement

The data supporting the conclusions of this article will be shared by request from any qualified investigator.

## Ethics Statement

The studies involving human participants were reviewed and approved by Ethics Committee of Shanghai Fifth People's Hospital Fudan University. The patients/participants provided their written informed consent to participate in this study.

## Author Contributions

DW, XCheng, and QD designed and supervised the study. FW and XChen collected the clinical and imaging data and blood samples. HS and YS tested the blood biomarkers. YS and XY analyzed the data and prepared the tables and figures. XCheng, YS, and XChen wrote the manuscript. All authors contributed to the article and approved the submitted version.

## Conflict of Interest

The authors declare that the research was conducted in the absence of any commercial or financial relationships that could be construed as a potential conflict of interest. The reviewer B-QZ declared a shared affiliation, though no other collaboration, with several of the authors YS, XC, FW, QD, XC, and DW to the handling editor.

## References

[1] Powers WJ Rabinstein AA Ackerson T Adeoye OM Bambakidis NC Becker K. Guidelines for the early management of patients with acute ischemic stroke: a guideline for healthcare professionals from the American heart association/American stroke association. Stroke. (2018) 49:e46–110. 10.1161/STR.0000000000000158 29367334

[2] Whiteley WN Emberson J Lees KR Blackwell L Albers G Bluhmki E. Risk of intracerebral haemorrhage with alteplase after acute ischaemic stroke: a secondary analysis of an individual patient data meta-analysis. Lancet Neurol. (2016) 15:925–33. 10.1016/S1474-4422(16)30076-X 27289487

[3] Lees KR Emberson J Blackwell L Bluhmki E Davis SM Donnan GA. Effects of alteplase for acute stroke on the distribution of functional outcomes: a pooled analysis of 9 trials. Stroke. (2016) 47:2373–9. 10.1161/STROKEAHA.116.013644 27507856 PMC5024752

[4] Hacke W Kaste M Fieschi C von Kummer R Davalos A Meier D. Randomised double-blind placebo-controlled trial of thrombolytic therapy with intravenous alteplase in acute ischaemic stroke (ECASS II). Second European-australasian acute stroke study investigators. Lancet. (1998) 352:1245–51. 10.1016/S0140-6736(98)08020-9 9788453

[5] Qureshi N. Tissue plasminogen activator for acute ischemic stroke. N Engl J Med. (1996) 334:1406. 10.1056/NEJM199605233342114 8614439

[6] De Meyer SF Stoll G Wagner DD Kleinschnitz C. von Willebrand factor: an emerging target in stroke therapy. Stroke. (2012) 43:599–606. 10.1161/STROKEAHA.111.628867 22180250 PMC4102321

[7] de Meyer SF Deckmyn H vanhoorelbeke K. von Willebrand factor to the rescue. Blood. (2009) 113:5049–57. 10.1182/blood-2008-10-165621 19318682

[8] Patzke J Favaloro EJ. Laboratory testing for von willebrand factor activity by glycoprotein ib binding assays (VWF:GPIb). Methods Mol Biol. (2017) 1646:453–60. 10.1007/978-1-4939-7196-1_33 28804847

[9] Plautz WE Raval JS Dyer MR Rollins-Raval MA Zuckerbraun BS Neal MD. ADAMTS13: origins, applications, and prospects. Transfusion. (2018) 58:2453–62. 10.1111/trf.14804 30208220

[10] Chen X Cheng X Zhang S Wu D. ADAMTS13: an emerging target in stroke therapy. Front Neurol. (2019) 10:772. 10.3389/fneur.2019.00772 31379722 PMC6650536

[11] de Meyer SF Savchenko AS Haas MS Schatzberg D Carroll MC Schiviz A. Protective15 anti-inflammatory effect of ADAMTS13 on myocardial ischemia/reperfusion injury in mice. Blood. (2012) 120:5217–23. 10.1182/blood-2012-06-439935 22915644 PMC3537313

[12] Khan MM Motto DG Lentz SR Chauhan AK. ADAMTS13 reduces VWF-mediated acute inflammation following focal cerebral ischemia in mice. J Thromb Haemost. (2012) 10:1665–71. 10.1111/j.1538-7836.2012.04822.x 22712744 PMC3419774

[13] Wang L Fan W Cai P Fan M Zhu X Dai Y. Recombinant ADAMTS13 reduces tissue plasminogen activator-induced hemorrhage after stroke in mice. Ann Neurol. (2013) 73:189–98. 10.1002/ana.23762 23280993

[14] Bath PM Blann A Smith N Butterworth RJ. Von Willebrand factor, P-selectin and fibrinogen levels in patients with acute ischaemic and haemorrhagic stroke, and their relationship with stroke sub-type and functional outcome. Platelets. (1998) 9:155–9. 10.1080/09537109876618 16793694

[15] Bustamante A Ning M Garcia-Berrocoso T Penalba A Boada C Simats A. Usefulness of ADAMTS13 to predict response to recanalization therapies in acute ischemic stroke. Neurology. (2018) 90:e995–1004. 10.1212/WNL.0000000000005162 29444972 PMC5874450

[16] Putzer AS Worthmann H Grosse GM Goetz F Martens-Lobenhoffer J Dirks M. ADAMTS13 activity is associated with early neurological improvement in acute ischemic stroke patients treated with intravenous thrombolysis. J Thromb Thrombolysis. (2019) 49:67–74. 10.1007/s11239-019-01941-7 31482326

[17] Hong L Cheng X Lin L Bivard A Ling Y Butcher K. The blood pressure paradox in acute ischemic stroke. Ann Neurol. (2019) 85:331–9. 10.1002/ana.25428 30720216

[18] Schuppner R Dirks M Grosse GM Bockmann M Goetz F Pasedag T. ADAMTS-13 activity predicts outcome in acute ischaemic stroke patients undergoing endovascular treatment. Thromb Haemost. (2018) 118:758–67. 10.1055/s-0038-1637732 29618156

[19] Haussen DC Jadhav A Jovin T Grossberg JA Grigoryan M Nahab F. Endovascular management vs intravenous thrombolysis for acute stroke secondary to carotid artery dissection: local experience and systematic review. Neurosurgery. (2016) 78:709–16. 10.1227/NEU.0000000000001072 26492430

[20] Lin J Liang Y Lin J. Endovascular therapy versus intravenous thrombolysis in cervical artery dissection-related ischemic stroke: a meta-analysis. J Neurol. (2020) 267:1585–93. 10.1007/s00415-019-09474-y 31321515

[21] Xu H Cao Y Yang X Cai P Kang L Zhu X. ADAMTS13 controls vascular remodeling by modifying VWF reactivity during stroke recovery. Blood. (2017) 130:11–22. 10.1182/blood-2016-10-747089 28428179 PMC5501147

[22] McCabe DJ Murphy SJ Starke R Harrison P Brown MM Sidhu PS Relationship between ADAMTS13 activity, von Willebrand factor antigen levels and platelet function in the early and late phases after TIA or ischaemic stroke. J Neurol Sci. (2015) 348:35–40. 10.1016/j.jns.2014.10.035 25498844

[23] Zhao BQ Chauhan AK Canault M Patten IS Yang JJ Dockal M. von Willebrand factor-cleaving protease ADAMTS13 reduces ischemic brain injury in experimental stroke. Blood. (2009) 114:3329–34. 10.1182/blood-2009-03-213264 19687510 PMC2759655

[24] Fujioka M Hayakawa K Mishima K Kunizawa A Irie K Higuchi S. ADAMTS13 gene deletion aggravates ischemic brain damage: a possible neuroprotective role of ADAMTS13 by ameliorating postischemic hypoperfusion. Blood. (2010) 115:1650–3. 10.1182/blood-2009-06-230110 19965676

[25] Yang S Jin M Lin S Cataland S Wu H. ADAMTS13 activity and antigen during therapy and follow-up of patients with idiopathic thrombotic thrombocytopenic purpura: correlation with clinical outcome. Haematologica. (2011) 96:1521–7. 10.3324/haematol.2011.042945 21606162 PMC3186314

